# Severe Hypertriglyceridaemia-Induced Necrotising Pancreatitis Complicated by Multi-organ Failure: A Case Report

**DOI:** 10.7759/cureus.111812

**Published:** 2026-06-30

**Authors:** Aharabie Jeyabavan, Guillaume Lambert, Nadeem M Sabir

**Affiliations:** 1 General Surgery, London North West University Healthcare NHS Trust, London, GBR; 2 Intensive Care Unit, London North West University Healthcare NHS Trust, London, GBR

**Keywords:** abdominal compartment syndrome (acs), hyperlipidemia, hypertriglyceridemia, necrotising pancreatitis, splenic vein thrombosis, systemic complications

## Abstract

We report a case of a woman in her 30s presenting with severe epigastric pain and vomiting. Initial laboratory analysis was significantly delayed due to marked lipaemia. Subsequent investigations revealed an elevated serum amylase (2636 IU/L) and markedly raised fasting triglycerides (20.4 mmol/L). Imaging confirmed acute pancreatitis, which progressed to necrotising pancreatitis complicated by splenic vein thrombosis and infected intra-abdominal collections. Her clinical course was further complicated by acute respiratory distress syndrome and abdominal compartment syndrome, necessitating prolonged intensive care admission. Management included haemofiltration, insulin infusion for triglyceride reduction, anticoagulation, broad-spectrum antimicrobials, and image-guided drainage of infected collections.

This case highlights the diagnostic and therapeutic challenges of hypertriglyceridaemia-induced pancreatitis and underscores the risk of significant complications and the importance of a multidisciplinary approach to such cases.

## Introduction

Acute pancreatitis is an inflammatory condition with a wide spectrum of severity. While gallstones and alcohol remain the most common causes, hypertriglyceridaemia is an important but under-recognised aetiology. The prevalence of hypertriglyceridaemia-induced pancreatitis is thought to account for about 5%-10% of cases of acute pancreatitis. The exact pathophysiology of hypertriglyceridaemia-induced pancreatitis is not completely understood. Proposed theories suggest hypertriglyceridaemia can lead to an increase in chylomicrons (lipoprotein particles composed mainly of triglycerides). This triggers various pathways, including hydrolysis of triglycerides into free fatty acids, leading to ischaemia and inflammation, which ultimately leads to the development of acute pancreatitis [1]. It is typically associated with more severe disease and a higher risk of complications [2]. Severe acute pancreatitis, as defined by the Revised Atlanta Classification, involves persistent organ failure lasting more than 48 hours and carries significant mortality [3]. Complications include pancreatic necrosis, systemic inflammatory response, and multi-organ dysfunction, and can carry a mortality of up to 40% [4]. 

We present a case of severe hypertriglyceridaemia-induced pancreatitis with a prolonged and complicated clinical course, highlighting diagnostic challenges and complex multidisciplinary management.

## Case presentation

A woman in her 30s of Filipino heritage and of normal body habitus presented to the emergency department with a two-day history of severe abdominal pain and multiple episodes of vomiting. She had a past medical history significant for irritable bowel syndrome, polycystic ovarian syndrome, and bipolar disorder. Her regular medications included mirtazapine, escitalopram, and eloine. There was no relevant family history (particularly no history of hypertriglyceridaemia or hypercholesterolaemia). She also had a history of significant alcohol intake, having consumed a large amount whilst on holiday a few weeks prior to her presentation.

On admission, clinical examination revealed epigastric tenderness with voluntary guarding. A bedside ultrasound was performed at this time, demonstrating a mildly swollen pancreatic head and hypoechoic areas within both the head and body of the pancreas. Her initial blood tests were lipaemic, delaying full interpretation. Following further analysis of the initial blood samples, a few values were obtained; the most notable were an amylase level of 2636 iu/L (normal 30-110), raised inflammatory markers, and raised creatinine, marking an initial Stage 1 acute kidney injury (AKI) that soon progressed to Stage 2 AKI.

She was admitted to the intensive treatment unit (ITU) for haemofiltration due to worsening fluid overload and progressing AKI. It was two days into her admission before her blood results could be interpreted and measured, showing a triglyceride level of 5.4 mmol/L and a fasting triglyceride of 20.4 mmol/L (normal range: 0.0-2.30 mmol/L) (Table 1). Contrast-enhanced CT imaging confirmed acute pancreatitis, which progressed to necrotising pancreatitis with splenic vein thrombosis and multiple intra-abdominal collections requiring percutaneous drainage.

**Table 1:** Initial blood tests (delayed interpretation)

Labs	Value (Unit)	Reference Range
White blood cells	10.9 (10^9/L)	3.0 - 10.0
Red blood cells	3.99 (10^12/L)	3.95 - 5.15
Haemoglobin	119 g/L	115 - 155
Neutrophil	8.7 (10^9/L)	2.0 - 7.5
Platelet count	263 (10^9/L)	150 - 400
Activated partial thromboplastin time blood	40.7 second(s)	26.3 - 36.6
Sodium	124 mmol/L	133 - 146
Potassium	4.0 mmol/L	3.5 - 5.3
Chloride	95 mmol/L	95 - 108
Urea	3.3 mmol/L	2.5 - 7.8
Creatinine	82 mmol/L	45 - 84
Alkaline phosphate	50 IU/L	30 - 130
Total bilirubin	73 umol/L	0 - 21
Alanine aminotransferase	37 IU/L	10 - 35
C-reactive protein level	363.0 mg/L	0 - 5
Glucose	9.2 mmol/L	3.4 - 6.0
HbA1c	40 mmol/mol	
Amylase	2636 IU/L	28 - 100
Triglycerides	5.3 mmol/L	0.0 - 2.3
Fasting triglycerides	20.4 mmol/L	0.0 - 2.3

Her condition deteriorated with the development of abdominal compartment syndrome (intra-abdominal pressure: 40 mmHg) and acute respiratory distress syndrome, necessitating mechanical ventilation and eventual tracheostomy. She was started on a variety of treatments to manage the pancreatitis, the underlying hypertriglyceridaemia, and other complications she developed.

Her management included (Table 2) intravenous insulin infusion for triglyceride reduction (Figure 1), anticoagulation for venous thromboses, broad-spectrum antimicrobial therapy guided by cultures, and interventional radiology drainage of infected collections.

**Table 2:** Timeline of interventions during the four-month admission

Phase	Treatment	Outcome
ED admission	CT of the abdomen and pelvis was completed, and blood samples were noted to be lipaemic. Started on IV saline	Acute pancreatitis confirmed
Discussion with the endocrine team	Start fixed-rate insulin at 0.05 units/kg/hour for hyperlipidaemia	Blood tests were repeated, but unreadable as samples remained lipaemic
Intensive treatment unit (ITU) admission	Arterial blood gas (ABG) analysis demonstrated worsening lactic acidosis, and blood samples remained lipaemic. Invasive monitoring was organised.	Demonstrated pancreatic necrosis and splenic vein thrombus (heparin infusion started)
Acute deterioration in the ITU, respiratory distress, the chest X-ray showed findings consistent with acute respiratory distress syndrome, abdominal pressure was 40 mmHg	Intubated and ventilated, started on hemofiltration and antibiotics escalated to meropenem	Aim to stabilise infection markers and ventilatory support
Endocrinology input	Unable to start fenofibrate due to deranged liver function, but could give ezetimibe via nasojejunal tube	Gradual reduction in triglyceride level. Level remained < 10, so fixed rate insulin infusion stopped
Repeat CT showing necrotic collections	Interventional radiology drainage of collections: high output from the drain, bilious output was 500 ml in less than 24 hours	Stabilising inflammatory markers
Extubated and tracheostomy inserted	Aiding in ventilatory weaning	Preparing the patient for stepdown to the ward
CT pulmonary angiography (CTPA) demonstrated thrombi in the superior vena cava and the internal jugular vein.	Heparin infusion, which was then changed to treatment with enoxaparin and then apixaban	Regular clotting profile checked to monitor the development of thrombocytopenia
Development of bilateral pleural effusions	Chest drain inserted	Improved respiratory effort and exercise threshold, and the drain was removed after four days
Stepped down to the ward	Ongoing abdominal drain output monitoring, antibiotics, and physiotherapy input	Discharged with surgical clinic follow-up for IV antibiotics and drain monitoring
Outpatient follow-up by the surgical team	The abdominal drain was removed, antibiotics course was completed	Discharged with lipid clinic and anticoagulation clinic follow-up

**Figure 1:**
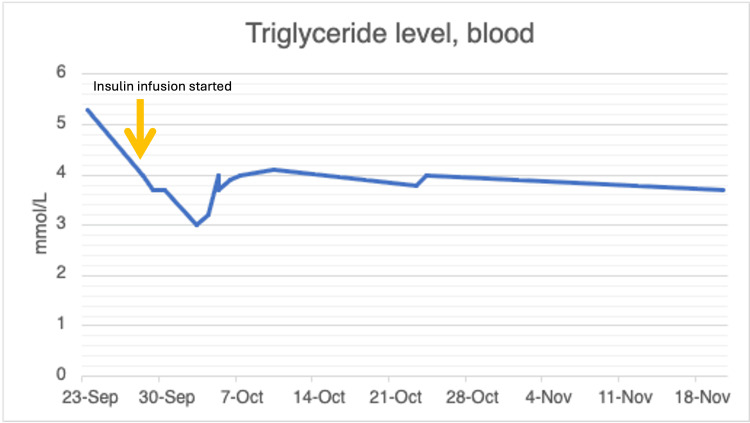
Graph to show trend in triglyceride levels during admission

Due to the multiple complications already posed by her pancreatitis, her case mandated numerous multidisciplinary meetings involving the intensive care team, the general surgical team, microbiology, haematology, and endocrinology teams. Surgical intervention was considered not advisable, and the severe acute pancreatitis was treated medically with ongoing input from all the teams mentioned above. 

Imaging

Imaging on admission confirmed acute pancreatitis with some abdominal free fluid (Figure 2). Due to worsening clinical state, contrast-enhanced CT imaging was repeated 48 hours later. This demonstrated necrotising pancreatitis complicated by splenic vein thrombosis (Figure 3). Ongoing interval scans demonstrated the development of intra-abdominal collections, which led to the insertion of two abdominal drains. 

**Figure 2:**
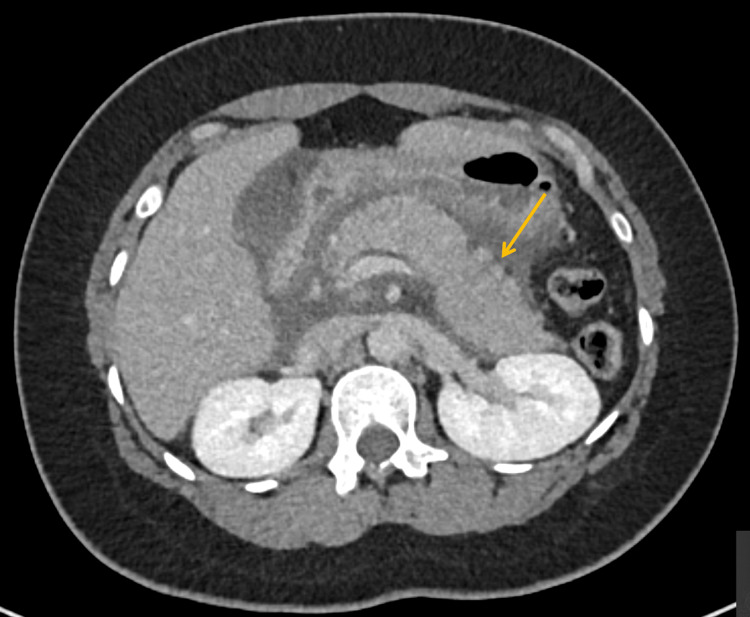
CT appearances indicative of acute pancreatitis affecting the pancreatic head and reactive descending duodenal mural oedema.

**Figure 3:**
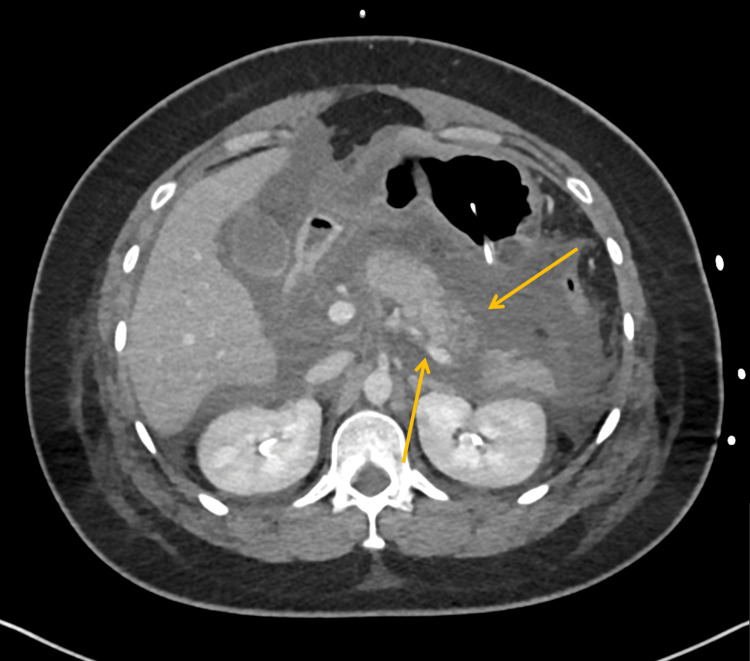
Progressive acute pancreatitis with evidence of necrosis at body/tail complicated by splenic vein thrombosis.

Outcome and follow-up

Following a four-month hospital admission, of which three months were spent in ITU, the patient was discharged with an abdominal drain in situ and regular follow-up appointments in the surgical acute care unit. She was discharged with bezafibrate and ezetimibe as maintenance for triglyceride management and apixaban for the splenic vein thrombosis. After one month, the abdominal drain was removed, as there was minimal output of serous fluid, and she completed an extensive course of antibiotics and antifungals. She was discharged from the surgical team with follow-up appointments in the lipid clinic and anticoagulation clinics. As per clinic letters, her lipid control has much improved with the abovementioned, with her lipid profile, including triglycerides (1.6 mmol/L), within the acceptable range of medications, and her pancreatic function has remained stable.

## Discussion

This case illustrates several important clinical challenges. 

Diagnostic delay due to hyperlipidaemia 

Initial blood samples were lipaemic, significantly hindering diagnosis. Lipaemic samples, characterised by markedly elevated triglyceride concentrations and the presence of chylomicrons, are a well-recognised source of laboratory interference and may obscure or delay the diagnosis of hypertriglyceridaemia-induced pancreatitis. Clinicians should therefore maintain a high index of suspicion when serum appears visibly turbid or milky in the appropriate clinical context. 

Lipaemia interferes with biochemical analysis through several mechanisms [5]. First, spectrophotometric interference occurs because excess lipid particles scatter incident light within automated analysers, leading to erroneous absorbance measurements and potentially causing falsely elevated or reduced analyte values depending on the assay methodology used. Second, severe lipaemia produces a volume displacement effect. Under normal physiological conditions, plasma consists of approximately 92% water; however, in grossly lipaemic samples, the lipid fraction may increase substantially, reducing the aqueous phase in which electrolytes are dissolved [6]. This may result in pseudohyponatraemia and falsely low electrolyte measurements [7]. Finally, lipoproteins may directly interfere with immunochemical and enzymatic assays by disrupting antigen-antibody interactions or altering reagent activity, thereby further compromising analytical accuracy. Recognition of these mechanisms is important, as laboratory artefacts caused by lipaemia can obscure biochemical interpretation and hinder timely clinical decision-making and possibly lead to the development of more severe and complicated disease progression.

Management of hypertriglyceridaemia 

A crucial aspect of managing this presentation of pancreatitis was treating the hypertriglyceridaemia. Insulin therapy was successfully utilised to reduce triglyceride levels. While plasmapheresis has been proposed as an alternative, its availability and evidence base remain limited, making insulin a practical first-line therapy in many centres [8].

Hypertriglyceridaemia is defined as a fasting triglyceride level greater than 1.7mmol/L [9]. Hypertriglyceridaemia is the third most common cause of acute pancreatitis and is typically associated with triglyceride levels exceeding 11.3 mmol/L. As mentioned, the exact pathophysiology is unclear [1]. However, there are two branches of aetiology to consider when understanding the relationship between high triglycerides and pancreatitis: genetic causes and secondary disorders of lipoprotein metabolism. Secondary causes of elevated triglycerides include alcohol and certain medications. This patient was known to have consumed large amounts of alcohol for many years prior to admission. Unfortunately, the association between these two factors is poorly understood. Although medications can account for a small proportion of acute pancreatitis cases, estimated at approximately 2%, there isn’t enough evidence to suggest medications as a causative factor here. While isolated reports suggest that mirtazapine‑induced hypertriglyceridaemia may increase pancreatitis risk, this association remains uncommon and poorly defined. In this case, the patient had been taking mirtazapine for six months prior to presentation without any preceding biochemical abnormalities or symptoms, making a direct drug‑related effect unlikely [10]. 

A literature review analysing 34 cases of hypertriglyceridaemia-induced pancreatitis demonstrated the effectiveness of insulin therapy. Similar to our case, insulin was utilised due to its ability to reduce serum triglyceride levels by activating the enzyme lipoprotein lipase and inhibiting hormone-sensitive lipase. Lipoprotein lipase promotes the breakdown of lipid-rich particles, chylomicrons and very low-density lipoproteins, into free fatty acids and glycerol, thereby lowering circulating triglyceride levels. Concurrently, inhibition of hormone-sensitive lipase reduces triglyceride breakdown within adipocytes (fat cells), decreasing the release of free fatty acids into the circulation. This may help mitigate their toxic effects on the pancreas and limit ongoing inflammation [11]. In severe hypertriglyceridaemia-induced pancreatitis, this dual action of insulin rapidly drops serum triglyceride levels, usually lowering them to a safe threshold. Therefore, insulin therapy was the effective non-invasive therapy to utilise in this case. 

Management of severe complications 

During her very long admission, this patient developed multiple complications. Initially, imaging revealed necrosis of the pancreas. This is a complication that occurs in 20% of acute pancreatitis cases, with a mortality rate of 15% and up to 40% if infection develops [4]. Multidisciplinary discussions were involved in the decision regarding the intervention for the necrotic collections that subsequently developed. It was decided to insert CT-guided drains in the collections. This approach has been shown to be effective in 56% of cases, without the need for alternative surgical interventions [12]. Within 24 hours of drain insertion, 500 ml of bilious fluid was collected due to enteric collections. Cultures guided antimicrobial management. Initial blood cultures grew *Enterobacter ludwigii *and *Staphylococcus epidermidis*, and antibiotics were escalated. Cultures from the intra-abdominal collections reiterated the growth of these organisms. Through guidance from the microbiologists, vancomycin and meropenem were stopped, and tigecycline was commenced due to the resistant nature of the bacteria grown. 

Followed shortly after this was the thrombosis of her splenic vessel noted on her CT scan, which is a common complication of pancreatitis, seen in about 20% of cases [13]. This pathology is thought to be due to surrounding pancreatic inflammation leading to compression of the splenic vessel and the hypercoagulable state, then causing the formation of a thrombus. Due to the increased risk of haemorrhage in pancreatitis, the use of anticoagulation is normally not recommended [14]. However, untreated splenic vein thrombosis in pancreatitis can lead to sinistral (left-sided) portal hypertension. Without intervention, this causes severe, localised complications like life-threatening gastrointestinal or variceal bleeding, enlarged spleen, and intestinal ischemia. Thus, it was decided to start a heparin infusion whilst closely monitoring her clotting profile and observing for any evidence of bleeding. 

Abdominal compartment syndrome is a known complication of pancreatitis with a mortality rate of 30%-40% [15]. Due to the ongoing inflammation and development of abdominal collections, the patient’s intra-abdominal pressure increased over the first few days following her admission. Abdominal compartment syndrome is defined as a sustained increase of intra-abdominal pressure greater than 20 mmHg associated with organ dysfunction. The large inflammatory response in pancreatitis causes third spacing of fluids in the abdomen as large volumes of administered fluids escape the bloodstream and pool in the bowel tissues and retroperitoneal space. This patient's intra-abdominal pressure significantly impacted her respiratory efforts, leading to significant respiratory distress. She was intubated and ventilated. Weaning her from the ventilator proved to be difficult due to the persistent high ventilatory pressures. Percutaneous drainage of the collections helped reduce the intra-abdominal hypertension. The decision was made that a tracheostomy would be the most suitable next step to aid weaning. This was challenging due to the high ventilatory pressures. She was successfully weaned off the ventilator and decannulated one week later. 

## Conclusions

Although gallstones and alcohol are the most common aetiologies, elevated triglyceride levels are an under-recognised but crucial cause of this pathology. This case highlights the significant morbidity associated with hypertriglyceridaemia-induced acute pancreatitis and the complexity of its management. It has demonstrated the difficulty in early recognition of the cause, particularly in the presence of very lipaemic samples. It emphasises how identification is essential to guide targeted therapy and demonstrates the effectiveness of insulin therapy in triglyceride reduction. Whilst it was determined that hypertriglyceridaemia was the driving cause of this case of severe pancreatitis, there remained uncertainty regarding the underlying cause of the elevated triglyceride levels. Additionally, the recent increased alcohol consumption in the weeks leading up to the presentation could not be excluded as a contributing factor. Her case was complicated by necrotising pancreatitis, venous thromboses and abdominal compartment syndrome, illustrating the wide-ranging systemic impact of the severe disease.

The management required a coordinated multidisciplinary approach, with treatment remaining primarily supportive through insulin infusions, fibrate and ezetimibe therapy and multiple interventional radiology-guided drains. The complex nature of this disease was further supported by the necessity of a prolonged intensive care admission, and microbiology-guided antibiotics and a structured multidisciplinary outpatient follow-up. 
